# Change in vasoactive inotropic score following hydrocortisone administration in the pediatric intensive care unit: a PICU data collaborative study, 2010–2022

**DOI:** 10.3389/fped.2026.1741717

**Published:** 2026-03-13

**Authors:** Colin M. Rogerson, Stephanie R. Brown, Colleen Badke, Adam Dziorny, Reid W. D. Farris, Tellen D. Bennett, Daniel Tawfik, Tim T. Cornell, Randall C. Wetzel, Akira Nishisaki, Julia A. Heneghan

**Affiliations:** 1 Department of Pediatrics, Division of Critical Care Medicine, Indiana University School of Medicine, Indianapolis, IN, United States; 2 Regenstrief Institute Center for Biomedical Informatics, Indianapolis, IN, United States; 3 Department of Pediatrics, Emory University School of Medicine, Atlanta, GA, United States; 4 Division of Critical Care Medicine, Children’s Healthcare of Atlanta, Atlanta, GA, United States; 5 Department of Pediatrics (Critical Care Medicine), Northwestern University Feinberg School of Medicine, Ann & Robert H. Lurie Children’s Hospital of Chicago, Chicago, IL, United States; 6 Department of Preventive Medicine (Health & Biomedical Informatics), Northwestern University Feinberg School of Medicine, Ann & Robert H. Lurie Children’s Hospital of Chicago, Chicago, IL, United States; 7 Department of Pediatrics (Critical Care Medicine), University of Rochester School of Medicine, Rochester, NY, United States; 8 Department of Biomedical Engineering, University of Rochester School of Medicine, Rochester, NY, United States; 9 Department of Pediatrics (Critical Care Medicine), University of Washington School of Medicine, Seattle Children’s Hospital, Seattle, WA, United States; 10 Department of Biomedical Informatics, University of Colorado School of Medicine, Aurora, CO, United States; 11 Department of Pediatrics (Critical Care), University of Colorado School of Medicine, Aurora, CO, United States; 12 Children’s Hospital Colorado, Aurora, CO, United States; 13 Department of Pediatrics (Critical Care Medicine), Stanford University School of Medicine, Lucile Packard Children’s Hospital Stanford, Palo Alto, CA, United States; 14 Laura P. and Leland K. Whittier Virtual Pediatric Intensive Care Unit, University of Southern California Keck School of Medicine, Children’s Hospital Los Angeles, Los Angeles, CA, United States; 15 Department of Anesthesia (Critical Care Medicine), University of Pennsylvania Perelman School of Medicine, Children’s Hospital of Philadelphia, Philadelphia, PA, United States; 16 Department of Pediatrics (Critical Care Medicine), University of Minnesota Medical School, Minneapolis, MN, United States

**Keywords:** critical care, informatics, pediatrics, resuscitation, shock

## Abstract

**Objective:**

Hydrocortisone (HCT) is an ancillary therapy for children with refractory hypotension. Current guidelines for HCT use are non-specific due to limited evidence of efficacy. We evaluated practice patterns and the association of HCT with the vasoactive inotropic score (VIS) in children admitted to the pediatric intensive care unit (PICU).

**Materials and methods:**

This was a retrospective observational cohort study using the PICU Data Collaborative (PDC). Our cohort consisted of PDC encounters receiving at least one of the six vasoactive medications included in the VIS calculation (epinephrine, norepinephrine, dopamine, dobutamine, vasopressin, or milrinone). We calculated hourly VIS for the first 7 days of ICU admission. We conducted a propensity score-matched comparison of change in VIS from hours 24–48 between encounters receiving HCT within the first 24 h of ICU admission and controls who did not receive HCT.

**Measurements and main results:**

The cohort included 10,244 encounters from three institutions. Of these, 1,915 (18.7%) received HCT. Encounters receiving HCT were older [5.4 (0.8–13.8) years vs. 4.3 (0.6–13.3) years, respectively] and had a higher maximum VIS [15.0 (10.0–20.2) vs. 8.0 (5.0–13.0)]. The PICU cohort had a significantly greater decrease in VIS from 24 to 48 h after admission compared with the cardiovascular intensive care unit (CVICU) cohort [82.2% (−40% to 100%) vs. 16.2% (−4.0% to 100%); *p* < 0.01]. In the matched PICU cohort, there was no difference in the 24-h reference VIS; however, there was a greater decrease in percentage of VIS from the 24-h reference to 48 h for those who received HCT [100.0 (50.0–100.0) vs. 86.6 (0.0–100.0); *p* = 0.03]. In the matched CVICU cohort, the HCT group had a higher VIS at 24 h [7.7 (1.0–11.0) vs. 6.0 (0.0–10.0); *p* = 0.02], with smaller decreases in VIS percent from reference at 48 h [9.3 (−13.2 to 58.7) vs. 18.2 (0.0–80.0); *p* = 0.01].

**Conclusions:**

The use of HCT was associated with greater improvement in VIS between 24 and 48 h after admission for children in the PICU, but not for children in the CVICU. Further research is needed to determine the optimal patient selection and timing of HCT use.

## Introduction

Hemodynamic support is a common reason for admission to the pediatric intensive care unit (PICU) (1). Initial management of inadequate hemodynamics includes intravenous fluid resuscitation and administration of vasoactive medications to maintain sufficient oxygen delivery to vital organs and tissues. While guidelines for hemodynamic support in this setting exist, current recommendations regarding ancillary pharmacologic therapies such as corticosteroids remain vague (2). Although corticosteroids can improve hemodynamics through up-regulation of *α*_1_ receptors and increased resorption of sodium and fluid, evidence of improved clinical outcomes with the use of corticosteroids is mixed.

In pediatrics, prior studies have shown that hydrocortisone (HCT) use is associated with worse outcomes in septic shock (3, 4). Adult studies, however, have reported no differences in vasoactive or ventilator-free days for patients receiving HCT (5), and contrarily found a reduction in short-term mortality and increased shock reversal at 7 days. Given these mixed results, it is likely that practice patterns regarding HCT vary widely in the PICU. However, neither this variability nor the association between HCT use and vasoactive requirements has been studied using a multi-institutional approach with highly granular electronic health record (EHR) data.

Our primary objective was to determine the association between HCT administration and change in vasoactive inotropic score (VIS) in a collaborative data network known as the PICU Data Collaborative (PDC) (6). Secondary objectives included evaluating the prevalence, timing, and frequency of HCT use in the PICU and pediatric cardiovascular intensive care unit (CVICU), as well as clinical outcomes including hospital length of stay (LOS), ICU LOS, and mortality.

## Materials and methods

### Overview

We conducted this retrospective observational cohort study using the PDC, which is a centralized network of eight academic children's hospitals contributing EHR data to a central repository (6). PDC data include PICU encounters and CVICU encounters from 2010 to 2022. All data were de-identified by the contributing institution prior to transmission to the PDC and underwent data quality and harmonization by the central data science team based at Children's Hospital Los Angeles. This study was approved by the PDC executive committee and determined to be non-human subject research by the Children's Healthcare of Atlanta Institutional Review Board (IRB #2677). Study details were reported in accordance with STROBE guidelines for observational research (7).

### Study population and data elements

Our study population included all encounters in the PDC that received continuous infusions of norepinephrine, epinephrine, dopamine, dobutamine, milrinone, and/or vasopressin, as these six medications are specifically included in the calculation of the VIS. Encounters lacking adequate medication data were excluded. For patients with multiple ICU admissions, only the first ICU admission was analyzed. Extracted data elements included demographics (age, sex, race, ethnicity), medications (dose, route, timing), and encounter information (ICU LOS, hospital LOS). Risk-of-mortality scores were not available; VIS was used as a marker of patient acuity. Vasoactive infusion data were measured each hour for the first 7 days (168 h) of ICU admission as weight-based dosing. Dosing gaps were imputed using a 6-h window with a last-observation-carried-forward technique up to a maximum of 6 h. An overall VIS was calculated for each hour of ICU admission up to 7 days (8). The dataset was extracted from the PDC in June 2024.

### Statistical analysis

Descriptive statistics were calculated as frequencies and percentages for categorical variables and tested using chi-square statistics. Continuous variables were measured as medians with interquartile ranges and compared using Wilcoxon Rank-Sum tests. The mean VIS was obtained for each hour following ICU admission. Total doses of HCT were counted, and timing of each was measured relative to ICU admission.

A propensity score-matched analysis was conducted based on the probability of each encounter to receive HCT. Eligible cases received HCT in the first 24 h of ICU admission. Eligible controls either received no HCT or received their first dose after 48 h in the ICU. Encounters with first HCT dose 24–48 h after ICU admission were excluded. Propensity scores were calculated using a logistic regression model that included site identifier, type of ICU (PICU vs. CVICU), sex, age, year, VIS at 24 h, and number of vasoactive infusions running at 24 h, with variables selected *a priori* based on clinical relevance. The model used exact matching for ICU type, a 1:1 ratio, and a 0.1 standard deviation caliper. We calculated Mahalanobis distance using age, year, VIS at 24 h, and number of vasoactive infusions running at 24 h (9). Average VIS was compared between matched cohorts, and percent change in VIS from the 24 h reference value through 48 h in 6-h intervals. We also conducted a planned sub-analysis based on ICU type. LOS was measured in days and compared using Wilcoxon Rank-Sum tests, and mortality was measured as frequency and compared using chi-square tests. We also conducted a sub-analysis of each cohort (PICU and CVICU) that received early HCT in the first 6 h after ICU admission and compared clinical outcomes (change in VIS percent at 48 h, hospital LOS, ICU LOS, and mortality) to those who received late HCT at 6–24 h after admission. All PDC data were housed in a Microsoft Azure environment, and data processing, image creation, and statistical tests were performed using embedded R and Python kernels (10). ChatGPT 3.0 and 40 mini were used as aids for code generation (11). Source code can be found here: https://github.com/sbrown513/Vasoactive-Inotrope-Score-Change-with-Hydrocortisone.

## Results

### Population

The cohort included 10,244 encounters receiving vasoactive medication infusions from three institutions. Five PDC institutions lacked adequate medication data at the time of data extraction. Of these encounters, 1,915 (18.7%) received HCT during ICU admission (Table 1). The HCT cohort had higher age [5.4 (0.8–13.8) vs. 4.3 (0.6–13.3)], higher maximum VIS [15.0 (10.0–20.2) vs. 8.0 (5.0–13.0)], longer hospital LOS [18.3 (9.1–36.5) vs. 10.8 (5.3–24.4)] and PICU LOS [6.3 (2.7–13.6) vs. 3.5 (1.7–7.9)], and increased mortality (21.7% vs. 10.0%). The PICU cohort had a significantly greater decrease in VIS from 24 to 48 h after admission compared with the CVICU cohort [82.2% (−40% to 100%) vs. 16.2% (−4.0% to 100%); *p* < 0.01].

**Table 1:** Cohort characteristics.

Characteristic	Whole cohort *N* = 10,244	Hydrocortisone *N* = 1,915
Age	4.3 (0.6–13.3)	5.4 (0.8–13.8)
Female (%)	4,628 (45.2)	864 (45.1)
Race (%)		
White	5,205 (50.8)	935 (48.8)
Black	550 (5.4)	100 (5.2)
Asian	1,029 (10.0)	168 (8.8)
Other	3,460 (33.8)	712 (37.2)
Ethnicity (%)		
Hispanic or Latino	2,842 (27.7)	578 (30.2)
Maximum VIS	8.0 (5.0–13.0)	15.0 (10.0–20.2)
Hospital LOS	10.8 (5.3–24.4)	18.3 (9.1–36.5)
PICU LOS	3.5 (1.7–7.9)	6.3 (2.7–13.6)
Mortality (%)	1,023 (10.0)	415 (21.7)

VIS, vasoactive inotropic score; LOS, length of stay.

Continuous statistics are presented as medians with interquartile ranges. Discrete variables are presented as counts with percentages.

### Hydrocortisone use

The median time from ICU admission to first HCT dose was 16.0 h (5.0–47.0), which varied widely between institutions (Table 2). Of encounters receiving HCT, 27.6% were receiving one vasoactive medication at time of first HCT dose, which was most frequently epinephrine (49.1%), dopamine (23.9%), or norepinephrine (19.6%), and 24.5% were receiving two vasoactive medications (Table 3). Stratifying by ICU type, at the time of HCT use, the PICU cohort most used epinephrine (34.7%) and norepinephrine (27.4%), while the CVICU cohort used epinephrine (70.7%), dopamine (46.4%), and milrinone (40.0%) most frequently. The PICU cohort started HCT at a higher median VIS. Of the 1,915 encounters receiving HCT, 67.1% received five or more doses of HCT during their encounter (Supplementary Table 1).

**Table 2:** Site comparison of full cohort.

Characteristic	Site 1 *N* = 3,987	Site 2 *N* = 1,875	Site 3 *N* = 4,382
HCT percent use in first 24 h of ICU admission	431 (10.8)	199 (10.6)	512 (11.7)
Timing of HCT use from PICU admission (h)	9.0 (3.0–50.0)	27.0 (12.0–55.0)	14.0 (4.0–42.0)
VIS at HCT start	9.0 (1.0–20.0)	0.0 (0.0–5.0)	10.0 (3.0–14.0)
Vasoactives in use at time of first HCT dose	1 (1–2)	0 (0–1)	2 (1–3)

VIS, vasoactive inotropic score; HCT, hydrocortisone.

Continuous statistics are presented as medians with interquartile ranges.

**Table 3:** Vasoactive usage at time of first hydrocortisone dose.

Vasoactive	Vasoactive(s) being administered at time of first HCT administration [n (%)]			Vasoactive dosage at time of first HCT administration [median (IQR)]		
	Full cohort *N* = 1,915	PICU *N* = 1,150	CVICU *N* = 765	Full cohort	PICU	CVICU
Norepinephrine	375 (19.6%)	315 (27.4%)	60 (7.8%)	0.10 (0.05–0.20)	0.10 (0.05–0.20)	0.07 (0.05–0.10)
Epinephrine	940 (49.1%)	399 (34.7%)	541 (70.7%)	0.06 (0.03–0.14)	0.12 (0.07–0.30)	0.05 (0.03–0.08)
Vasopressin	221 (11.5%)	88 (7.7%)	133 (17.4%)	0.30 (0.01–0.50)	0.20 (0.01–0.50)	0.30 (0.20–0.50)
Milrinone	329 (17.2%)	23 (2.0%)	306 (40.0%)	0.50 (0.25–0.50)	0.25 (0.25–0.50)	0.50 (0.25–0.50)
Dopamine	457 (23.9%)	102 (8.9%)	355 (46.4%)	5.0 (5.0–6.0)	8.0 (5.0–10.0)	5.0 (5.0–5.0)
Dobutamine	4 (0.2%)	4 (0.3%)	0 (0.0%)	9.25 (5.0–13.13)	9.25 (5.0–13.13)	NA
				VIS Score at hydrocortisone start [median (IQR)]		
One vasoactive	528 (27.6%)	374 (32.5%)	154 (20.1%)	7.0 (4.0–13.0)	8.0 (5.0–15.0)	5.0 (3.3–8.0)
Two vasoactives	469 (24.5%)	215 (18.7%)	254 (33.2%)	13.6 (9.0–26.2)	26.0 (15.0–48.5)	10.0 (8.0–13.3)
Three vasoactives	268 (14.0%)	37 (3.2%)	231 (30.2%)	13.0 (11.0–16.0)	52.0 (27.0–75.0)	13.0 (11.0–15.7)
Four vasoactives	14 (0.7%)	4 (0.3%)	10 (1.3%)	22.3 (14.4–46.7)	58.5 (47.8–154.1)	17.8 (13.6–24.3)

All vasoactive doses presented in mcg/kg/min apart from vasopressin, which is presented in milliunits/kg/min.

### Propensity score comparison

**Figure 1:**
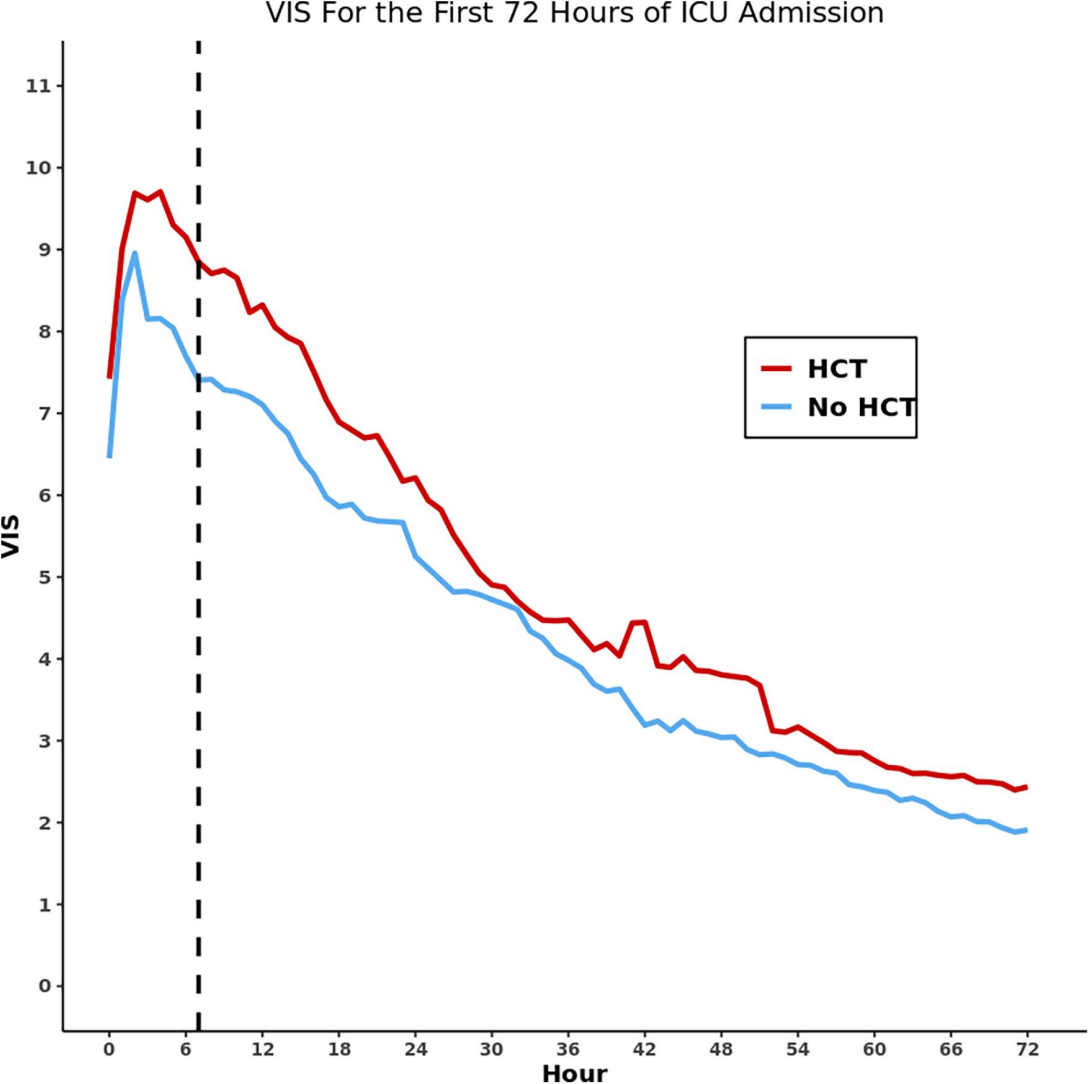
Line graph of vasoactive inotropic score over the first 72 h of ICU stay for the entire propensity score-matched cohort. The dashed vertical line represents the median timing of first hydrocortisone dose for each matched cohort.

**Figure 2:**
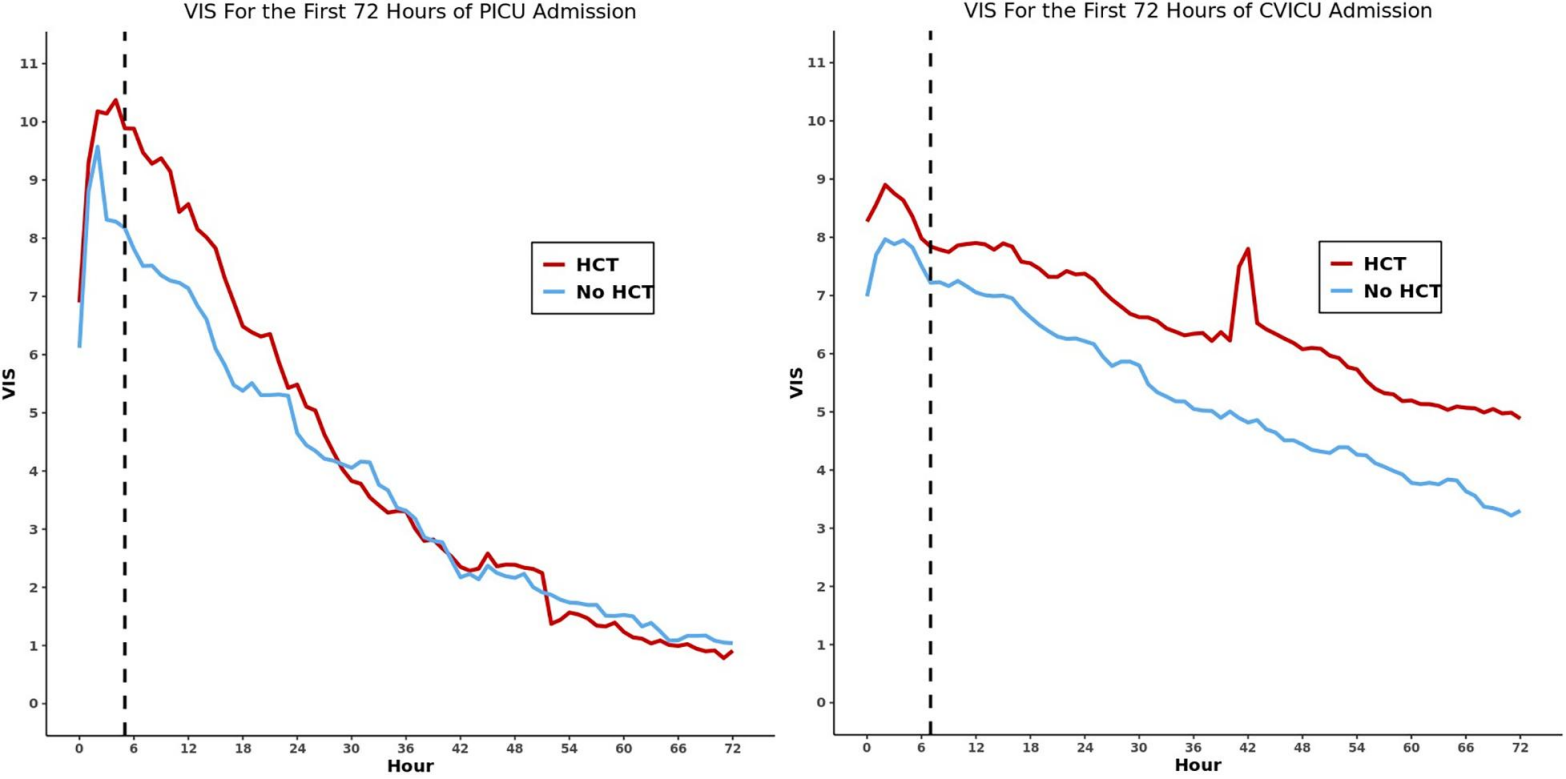
Line graphs of vasoactive inotropic score over the first 72 h of PICU stay (left) and CVICU stay (right) for the propensity score-matched cohorts. The dashed vertical line represents the median timing of first hydrocortisone dose for each matched cohort.

Using propensity scores, we matched 1,062 (93.0%) encounters of the 1,142 receiving HCT in the first 24 h of ICU admission (Supplementary Table 2). There was no significant difference in VIS at the 24-h reference point and no significant difference in VIS change from 24 to 48 h (Supplementary Table 3, Figure 1). In the sub-analysis based on ICU type, of the 654 matched PICU encounters, there was no difference in the 24-h reference VIS; however, there was a significantly greater decrease in percentage of VIS from the 24-h reference at three of four measured intervals (Table 4, Figure 2). Among the 408 matched CVICU encounters, the HCT group had significantly higher VIS at 24 h and a significantly smaller decrease in VIS percent at three of four measured intervals.

**Table 4:** Changes in VIS following hydrocortisone administration.

Timing	PICU cohort			CVICU cohort		
	Hydrocortisone (*n* = 654)	No hydrocortisone (*n* = 654)	*p*-value	Hydrocortisone (*n* = 408)	No hydrocortisone (*n* = 408)	*p*-value
24-h VIS (REF)	0.0 (0.0–5.0)	0.0 (0.0–5.0)	0.77	7.7 (1.0–11.0)	6.0 (0.0–10.0)	0.02^a^
	Percent decrease in VIS from reference			Percent decrease in VIS from reference		
30 h	25.7 (0.0 to 78.9)	14.8 (−27.4 to 60.0)	0.03^a^	0.0 (0.0 to 19.1)	0.0 (0.0 to 20.7)	0.61
36 h	61.5 (0.0 to 100.0)	41.9 (−25.0 to 100.0)	<0.01^a^	0.0 (−4.6 to 35.6)	0.0 (0.0 to 54.5)	0.047^a^
42 h	80.0 (20.0 to 100.0)	69.4 (0.0 to 100.0)	0.07	3.8 (−13.3 to 41.0)	14.1 (0.0 to 74.4)	<0.01^a^
48 h	100.0 (50.0 to 100.0)	86.6 (0.0 to 100.0)	0.03^a^	9.3 (−13.2 to 58.7)	18.2 (0.0 to 80.0)	0.01^a^

VIS, vasoactive inotropic score.

Values are measured as medians with interquartile ranges. Propensity score matching was performed using 1:1 nearest neighbor matching without replacement, with a caliper width of 0.1 standard deviation of the logit of the propensity score. Covariates included exact matching of ICU type, and non-exact matching of age, sex, year of admission, VIS at 24 h, and number of vasoactive medications running at 24 h after ICU admission. Covariate balance was assessed using standard mean differences, all of which were <0.1, indicating adequate balance.

a Statistically significant at an alpha level of 0.05.

Comparing LOS between the HCT and non-HCT cohorts, the PICU cohort that received HCT had a significantly shorter hospital LOS [11.8 days (4.9–26.4) vs. 14.8 days (7.5–31.1); *p* < 0.01] with no significant difference in ICU LOS [3.9 days (1.7–9.4) vs. 3.5 days (1.8–7.1); *p* = 0.21] (Supplementary Table 4). The CVICU cohort that received HCT had a significantly longer hospital LOS [20.1 days (12.1–37.7) vs. 15.3 days (6.9–32.1); *p* < 0.01] and a significantly longer ICU LOS [9.1 days (4.5–19.7) vs. 5.1 days (2.9–9.6); *p* < 0.01]. The PICU cohort that received HCT had no significant difference in mortality [*n* = 131 (20.0%) vs. *n* = 135 (20.6%); *p* = 0.84], while the CVICU cohort receiving HCT had significantly higher mortality [*n* = 66 (16.2%) vs. *n* = 10 (2.5%); *p* < 0.01].

The sub-analysis of early versus late HCT administration identified 334 (51.1%) encounters receiving HCT in the first 6 h of PICU admission, and 167 (40.9%) in the first 6 h of CVICU admission (Supplementary Table 5). There was no significant difference in percent decrease in VIS at 48 h in the PICU cohort [100% (42.9%–100%) vs. 100% (51.4%–100%); *p* = 0.35] or the CVICU cohort [15.4% (11.8%–57.8%) vs. 9.1% (−14.0%–58.6%); *p* = 0.63]. There were also no significant differences in hospital LOS for the early HCT administration group in the PICU [11.7 days (4.8–24.7) vs. 12.3 days (5.3–27.6); *p* = 0.62] or the CVICU [20.8 days (11.9–38.1) vs. 19.8 days (12.6–37.6); *p* = 0.82], or in ICU LOS for the PICU cohort [3.5 days (1.6–9.0) vs. 4.1 days (1.8–9.9); *p* = 0.29] or the CVICU cohort [9.1 days (3.9–20.0) vs. 9.0 days (4.7–18.8); *p* = 0.59]. There was a significant decrease in mortality rate for the early HCT cohort in the PICU [54/334 (16.2%) vs. 77/320 (24.1%); *p* = 0.02], but not in the CVICU [30/167 (18.0%) vs. 36/241 (14.9%); *p* = 0.50].

## Discussion

In this multicenter observational study of children receiving vasoactive infusions, we described practice patterns in vasoactive use surrounding HCT administration. We found that in PICU encounters, HCT use was associated with a more rapid decrease in VIS. For CVICU encounters, HCT use was associated with less rapid decrease in VIS over the subsequent 24 h.

The peak VIS and choice of vasoactive agent at the time of HCT use likely reflect current practice based on the Surviving Sepsis Guidelines, which recommend HCT for patients in septic shock who are refractory to vasoactive therapy (2). Our data may show general thresholds where ICU clinicians choose to define a patient as “refractory,” although these thresholds appear to vary by institution, with one site starting HCT further from PICU admission and with less vasoactive requirement, and two sites starting relatively quickly after PICU admission with higher vasoactive requirements. Further research is needed to establish optimal specific thresholds to improve clinical outcomes. An important consideration in our data is the presence of encounters where HCT was given prior to the initiation of vasoactive medications. This could be either due to data issues where HCT was ordered prior to the recording of vasoactive infusions or could be due to use of HCT for other indications such as adrenal insufficiency. We also chose to use ICU admission as time 0 rather than initial HCT administration. This adds some bias as encounters receiving HCT 1 h after admission will likely have different effects at 48 h than those who received HCT at 23 h after admission. However, there are many other ICU therapies for septic shock that are likely being given at ICU admission that cannot be accounted for in our study and would add bias if we chose HCT administration as time 0.

We found differential association of HCT use and VIS change based on the ICU type. Most PICU encounters with shock suffer primarily from distributive shock (12), with the initial treatment being fluid resuscitation and vasoconstriction through *α*_1_ agonism. HCT can potentiate these effects while also retaining intravascular fluid (13), which directly opposes the decreased systemic venous resistance (SVR) seen in distributive shock, which could explain our findings. We also found that the peak VIS was higher in the HCT group, which is intuitive as PICU clinicians generally reserve HCT until vasoactive infusions have proven unable to maintain adequate hemodynamics (4). This could mathematically favor the HCT group's VIS decrease by providing a higher peak to decrease from, and the rate of VIS decline equalizes in both groups after approximately 30 h. Moreover, we observed an association between HCT use and decreased hospital LOS in the PICU cohort, consistent with the VIS findings. This evidence suggests that there may be early hemodynamic benefits for PICU patients.

CVICU encounters experiencing shock are more likely to have cardiogenic shock. While HCT does have some effects on calcium regulation and beta-adrenergic receptors, it primarily functions by increasing SVR (13). While this may increase a patient's blood pressure, the additional afterload on the left ventricle may impair the cardiac output in these patients and lead to overall decreased oxygen delivery. Given to these considerations, it is unsurprising that the weaning trajectories for this patient population were different from those of the general PICU population. While prior work found no difference in the rate of VIS change with HCT use in the CVICU (14), our study actually found that CVICU patients who received HCT had a decreased rate of improvement in vasoactive requirements compared with those who did not receive HCT. This finding is most likely due to selection bias in our study, with the more acutely ill patients receiving HCT therapy to improve poor hemodynamics. It is theoretically possible though that increased SVR from HCT worsens cardiac output in this population and impairs clinicians' ability to wean vasoactive or inotropic support. The use of milrinone also may compound the complexity of this observation, as it reduces afterload and provides substantial benefit for cardiac patients but is rarely used in septic shock as it causes vasodilation. Milrinone's incorporation into the VIS can complicate our findings because its use will increase the VIS while potentially being a sign of recovery in some CVICU patients. We also found HCT use in the CVICU is associated with longer ICU and hospital LOS, and higher mortality rate, consistent with lack of improvement in VIS. Taken together, these results add to the growing body of literature suggesting heterogeneity of treatment effect for HCT in pediatrics, underscoring the need for further research to determine which patients are most likely to benefit (15–17).

There are theoretical benefits to earlier HCT administration relative to later administration, as patients may have a window of opportunity for HCT effects to counteract septic shock. In the present study, early versus late HCT use was not associated with differences in VIS change, ICU LOS. However, there was a significant association of early HCT use in the PICU cohort and decreased mortality. It is unclear why this association was absent with other clinical outcome benefits, but it likely warrants further investigation in other multicenter trials.

Our study has several strengths, including the large sample size and multi-institutional nature of the data contained in the PDC, as well as the ability to calculate hourly VIS values for a large population of critically ill patients. To our knowledge, no previous studies have been able to propensity score match this number of critically ill pediatric patients based on granular EHR elements such as number of vasoactive infusions and VIS scores to study the effects of HCT use. Despite these strengths, our study also has several weaknesses. Patient selection for shock is difficult due to lack of gold-standard diagnostic criteria, and our inclusion of all patients receiving vasoactive therapy may introduce bias. Our study is also subject to indication bias, as we cannot determine conclusively whether HCT was administered for a specific type of shock or for a different indication such as acute or chronic adrenal insufficiency. Vasoactive selection, dosing, and escalation are also subject to physician or institutional practices that may vary widely. Our data were unable to measure fluid resuscitation, which is an important factor in vasoactive usage. The use of VIS for encounter acuity without risk-of-mortality scores may impair patient matching. Data from continuous medications are imperfect and frequently contains gaps. We attempted to mitigate this using a last-observation-carried-forward imputation, but this may cause inaccurate VIS calculation. Our study population was heterogeneous, encompassing patients from both the PICU and CVICU, which have significantly different pathophysiologies. This heterogeneity likely explains much of the variability observed in weaning trajectories. While we attempted to leverage the entire PDC across eight sites, only three sites had adequate data to complete the analysis, limiting our ability to analyze variation in institutional practice patterns of HCT use. The use of extracorporeal membrane oxygenation therapy would affect the VIS for this patient population but was unavailable in the data for this study.

## Conclusion

The use of HCT in children on vasoactive infusions was common, occurred relatively early during the ICU encounter, and was associated with faster improvement in VIS among children in the PICU, but not among children in the CVICU. Further research is needed to determine the optimal patient selection and timing of HCT use.

## Data Availability

The datasets presented in this article are not readily available because Data for this study is housed in a secure, anonymized, cloud-based database that is not available for download. Requests to access the datasets should be directed to pdcadmin@vpicu.net.
