# Supplementary material for: Change in vasoactive inotropic score following hydrocortisone administration in the pediatric intensive care unit: a PICU data collaborative study, 2010–2022
Source: Front Pediatr. 2026 Mar 13;14:1741717. doi: 10.3389/fped.2026.1741717 (PMC13021878; doi:10.3389/fped.2026.1741717)
Supplement: Supplementary file 1 [file Supplementaryfile1.docx]

Supplemental Content

Table of Contents:

1: Supplemental Table 1 – page 2

2: Supplemental Table 2 – page 3-4

3: Supplemental Table 3 – page 5

4: Supplemental Table 4 – page 6

5: Supplemental Table 5 – page 7

Supplemental Table 1: Number of Hydrocortisone doses during PICU encounter

| **Doses** | **Frequency** |
| --- | --- |
| 1 | 1,915 (18.7%) |
| 2 | 1,730 (16.9%) |
| 3 | 1,533 (15.0%) |
| 4 | 1,412 (13.8%) |
| 5+ | 1,285 (12.5%) |

Supplemental Table 2: Matched Cohort Characteristics

| **Characteristic** | **Hydrocortisone**  **N=1,062** | **No Hydrocortisone**  **N=1,062** | **p-value** |
| --- | --- | --- | --- |
| Age | 6.4 (1.2-14.5) | 6.0 (1.0-14.3) | 0.49 |
| Female (%) | 498 (46.9) | 499 (47.0) | 1.0 |
| Race (%) |  |  |  |
| White | 499 (47.0) | 522 (49.2) |  |
| Black | 55 (5.2) | 53 (5.0) |  |
| Asian | 101 (9.5) | 107 (10.1) |  |
| Other | 407 (38.3) | 380 (35.8) | 0.65 |
| Ethnicity (%) |  |  |  |
| Hispanic or Latino | 325 (30.6) | 306 (28.8) | 0.39 |
|  |  |  |  |
| Site 1 | 388 (36.5) | 395 (37.2) |  |
| Site 2 | 194 (18.3) | 209 (19.7) |  |
| Site 3 | 480 (45.2) | 458 (43.1) | 0.57 |
|  |  |  |  |
| Year of admission |  |  |  |
| 2010 | 15 (1.4) | 13 (1.2) |  |
| 2011 | 17 (1.6) | 18 (1.7) |  |
| 2012 | 41 (3.9) | 39 (3.7) |  |
| 2013 | 49 (4.6) | 45 (4.2) |  |
| 2014 | 86 (8.1) | 92 (8.7) |  |
| 2015 | 100 (9.4) | 98 (9.2) |  |
| 2016 | 119 (11.2) | 119 (11.2) |  |
| 2017 | 134 (12.6) | 142 (13.4) |  |
| 2018 | 149 (14.0) | 138 (13.0) |  |
| 2019 | 151 (14.2) | 150 (14.1) |  |
| 2020 | 103 (9.7) | 112 (10.5) |  |
| 2021 | 57 (5.4) | 64 (6.0) |  |
| 2022 | 41 (3.9) | 32 (3.0) | 0.99 |
|  |  |  |  |
| PICU | 654 (61.6) | 654 (61.6) |  |
| CVICU | 408 (38.4) | 408 (38.4) | 1.0 |
|  |  |  |  |
| VIS at 24 hours | 5.8 (1.4-10.3) | 5.0 (1.4-9.2) | *0.02 |
| Vasoactive Count at 24 hours | 2 (1-2) | 2 (1-2) | 0.83 |
|  |  |  |  |
| Maximum VIS | 14.5 (9.0-25.0) | 12.0 (8.0-19.0) | *<0.01 |
| Hospital LOS | 16.2 (7.8-34.4) | 11.0 (5.9-23.3) | *<0.01 |
| PICU LOS | 5.8 (2.1-12.7) | 4.1 (2.0-8.0) | *<0.01 |
| Mortality (%) | 197 (18.5) | 145 (13.7) | *<0.01 |

VIS: Vasoactive Inotropic Score; LOS: Length of Stay

Continuous statistics are presented as medians with interquartile ranges

Discrete variables are presented as counts with percentages

*p-value <0.05 calculated with chi-square test for categorical variables or Mann-Whitney U test for continuous variables

Propensity score matching was performed using 1:1 nearest neighbor matching without replacement, with a caliper width of 0.1 standard deviation of the logit of the propensity score. Covariates included exact matching of ICU type, and non-exact matching of age, sex, year of admission, VIS at 24 hours, and number of vasoactive medications running at 24 hours after ICU admission. Covariate balance was assessed using standard mean differences, all of which were <0.1, indicating adequate balance.

Supplemental Table 3: Changes in VIS of cohort following Hydrocortisone

administration for the full cohort

| **Timing** | **Hydrocortisone**  **(n=1,062)** | **No Hydrocortisone**  **(n=1,062)** | **p-value** |
| --- | --- | --- | --- |
| 24-Hour VIS (REF) | 1.0 (0.0-9.5) | 0.1 (0.0-8.0) | 0.14 |
|  | **Percent Decrease in VIS from Reference** | |  |
| 30-Hours | 0.0 (0.0-0.50) | 0.0 (0.0-40.0) | 0.28 |
| 36-Hours | 16.9 (0.0-88.8) | 16.7 (0.0-85.7) | 0.69 |
| 42-Hours | 28.6 (0.0-100.0) | 33.3 (0.0-100.0) | 0.39 |
| 48-Hours | 47.0 (0.0-100.0) | 44.0 (0.0-100.0) | 0.90 |

VIS: Vasoactive Inotropic Score

Values are measured as medians with interquartile ranges

* Statistically significant at an alpha level of 0.05

Propensity score matching was performed using 1:1 nearest neighbor matching without replacement, with a caliper width of 0.1 standard deviation of the logit of the propensity score. Covariates included exact matching of ICU type, and non-exact matching of age, sex, year of admission, VIS at 24 hours, and number of vasoactive medications running at 24 hours after ICU admission. Covariate balance was assessed using standard mean differences, all of which were <0.1, indicating adequate balance.

Supplemental Table 4: LOS and Mortality Comparison of the Matched Cohorts

| **Cohort** | **Hospital LOS (days)** | **p** | **ICU LOS (days)** | **p** | **Mortality** | **p** |
| --- | --- | --- | --- | --- | --- | --- |
| PICU HCT (n=654) | 11.8 (4.9-26.4) | *<0.01 | 3.9 (1.7-9.4) | 0.21 | 131 (20.0%) | 0.84 |
| PICU No HCT (n=654) | 14.8 (7.5-31.1) |  | 3.5 (1.8-7.1) |  | 135 (20.6%) |  |
| CVICU HCT (n=408) | 20.1 (12.1-37.7) | *<0.01 | 9.1 (4.5-19.7) | *<0.01 | 66 (16.2%) | *<0.01 |
| CVICU No HCT  (n=408) | 15.3 (6.9-32.1) |  | 5.1 (2.9-9.6) |  | 10 (2.5%) |  |

* Statistically significant at an alpha level of 0.05

ICU: Intensive Care Unit

PICU: Pediatric Intensive Care Unit

CVICU: Cardiovascular Intensive Care Unit

HCT: Hydrocortisone

LOS: Length of Stay

Supplemental Table 5: Clinical Outcomes of Early vs. Late Hydrocortisone Use

| **Cohort** | **Change in VIS at 48 hours (%)** | **p** | **Hospital LOS (days)** | **p** | **ICU LOS (days)** | **p** | **Mortality** | **p** |
| --- | --- | --- | --- | --- | --- | --- | --- | --- |
| Early PICU (n=334) | 100% (42.9%-100%) | 0.35 | 11.7 (4.8-24.7) | 0.62 | 3.5 (1.6-9.0) | 0.29 | 54 (16.2%) | *0.02 |
| Late PICU (n=320) | 100% (51.4%-100%) |  | 12.3 (5.3-27.6) |  | 4.1 (1.8-9.9) |  | 77 (24.1%) |  |
| Early CVICU (n=167) | 15.4% (11.8%-57.8%) | 0.63 | 20.8 (11.9-38.1) | 0.82 | 9.1 (3.9-20.0) | 0.59 | 30 (18.0%) | 0.50 |
| Late CVICU (n=241) | 9.1% (-14.0%-58.6%) |  | 19.8 (12.6-37.6) |  | 9.0 (4.7-18.8) |  | 36 (14.9%) |  |

* Statistically significant at an alpha level of 0.05

Early is defined as receiving hydrocortisone within the first 6 hours of ICU admission. Late is defined as receiving hydrocortisone in 6-24 hours after ICU admission.

ICU: Intensive Care Unit

PICU: Pediatric Intensive Care Unit

CVICU: Cardiovascular Intensive Care Unit

VIS: Vasoactive Inotropic Score

LOS: Length of Stay
